# Predominantly Solid Hemangioblastoma Presenting as an Extra-Axial Cerebellopontine Angle Lesion

**DOI:** 10.7759/cureus.13071

**Published:** 2021-02-02

**Authors:** Gasim Ahmed, Usman Sheikh, Souhyb Masri, Jacob Joseph, Hemant Sonwalker

**Affiliations:** 1 Radiology, Lancashire Teaching Hospital Foundation Trust, Preston, GBR; 2 Radiology, The Christie NHS Foundation Trust, Manchester, GBR; 3 Pathology, Lancashire Teaching Hospital Foundation Trust, Preston, GBR

**Keywords:** hemangioblastoma, cerebellopontine, extra-axial, dural

## Abstract

Hemangioblastomas (HBs) are typically intra-axial, highly vascular tumors of the central nervous system and account for up to 2.5% of all intracranial tumors and up to 12% of posterior fossa neoplasms. Extra-axial HBs are rarely described in the literature. The radiological appearances of cerebellopontine angle (CPA) extra-axial HB can lead to a diagnostic conundrum as they may mimic the appearance of dural metastasis, vestibular schwannoma, or meningioma. Here, we describe a patient who presented with an extra-axial CPA HB and explore the literature of the condition.

## Introduction

Hemangioblastomas (HBs) are well‐differentiated, vascular, benign World Health Organization (WHO) grade I tumors that compromise 1.5% to 2.5% of all intracranial tumors and, in adults, 7% to 12% of all intra‐axial posterior fossa tumors. HBs of the cerebellopontine angle (CPA) are extremely rare with only a few cases being published in the literature. When present in the CPA, HBs exhibit extra-axial imaging characteristics leading to a diagnostic conundrum as their imaging appearance shares similarities with other CPA common tumors. Correct diagnosis is of pivotal importance due to the significant blood loss that may result from internal debulking of a misdiagnosed lesion.

## Case presentation

A 64-year-old right-handed Caucasian male with no significant medical history presented with a three-month complaint of an intermittent dull headache, vertigo, and unsteadiness. Physical examination was unremarkable.

**Figure 1:**
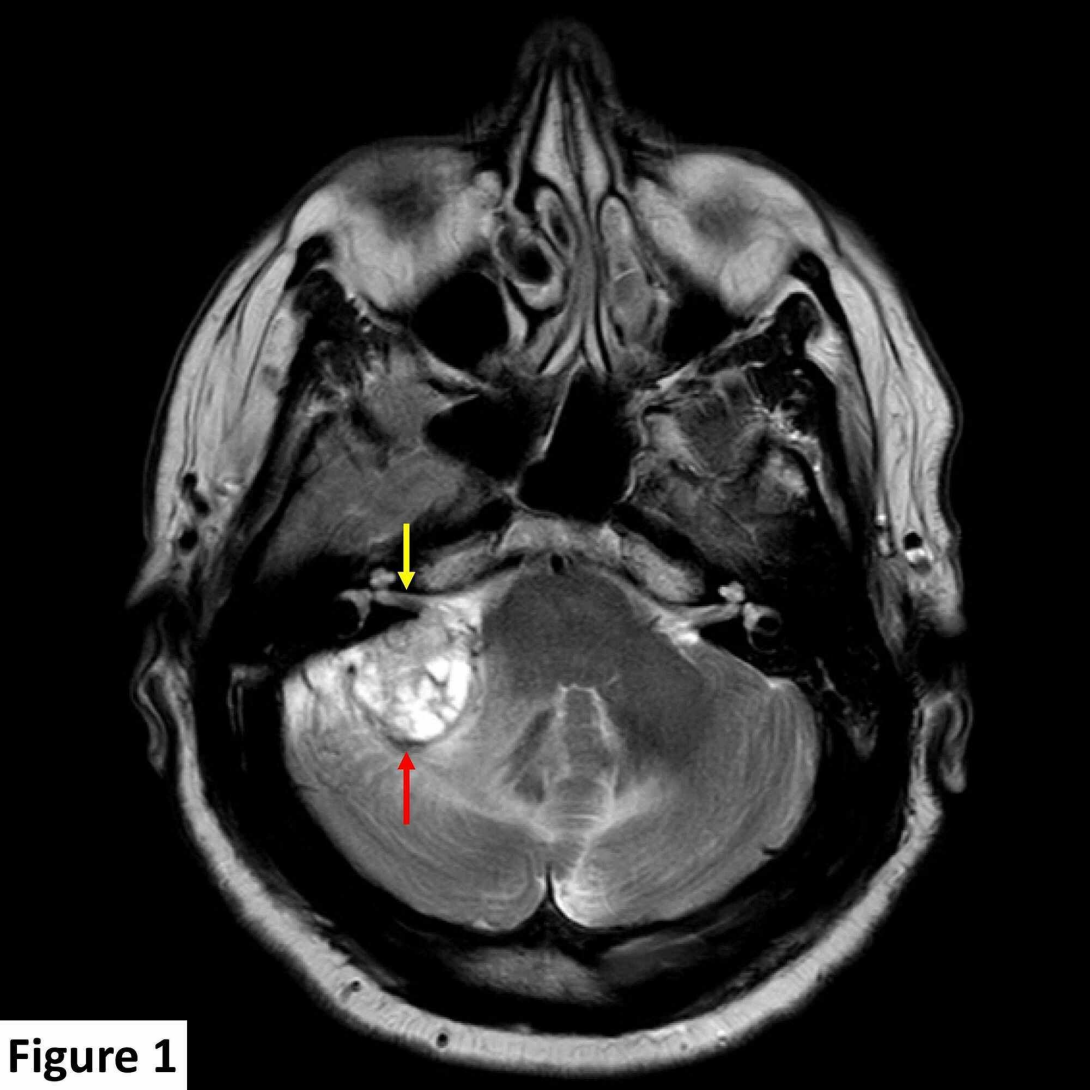
MRI scan at presentation. Axial T2 image showing a heterogenous T2 hyperintense lesion (red arrow) in the right cerebellopontine angle compressing the right middle cerebral peduncle, cerebellum, and fourth ventricle, with associated parenchymal high T2 signal change. The right internal auditory canal is spared (yellow arrow). MRI, magnetic resonance imaging

**Figure 2:**
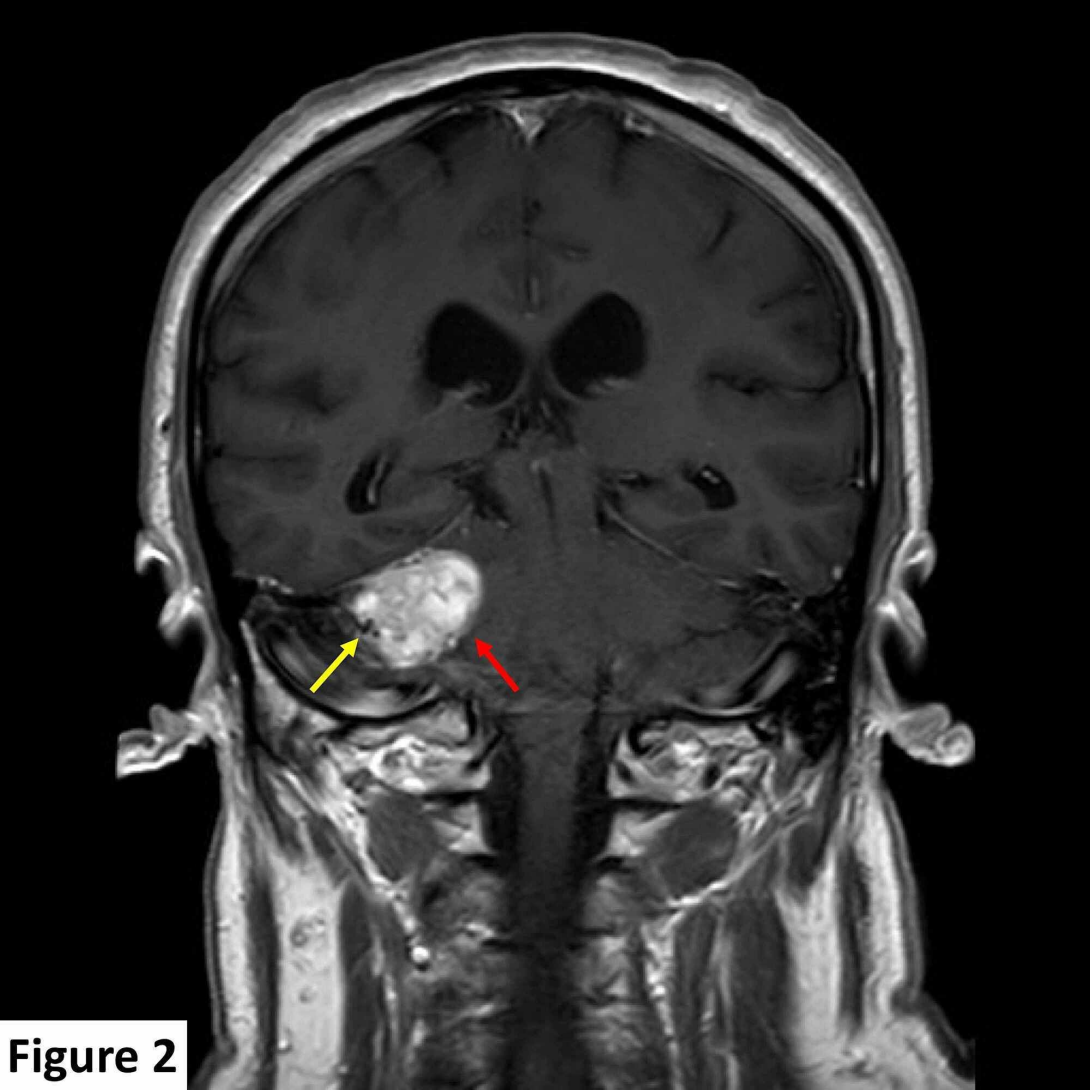
MRI scan at presentation. Coronal T1 contrast-enhanced image shows avid lesional enhancement (red arrow) and vascular flow void at its posterolateral aspect (yellow arrow). MRI, magnetic resonance imaging

**Figure 3:**
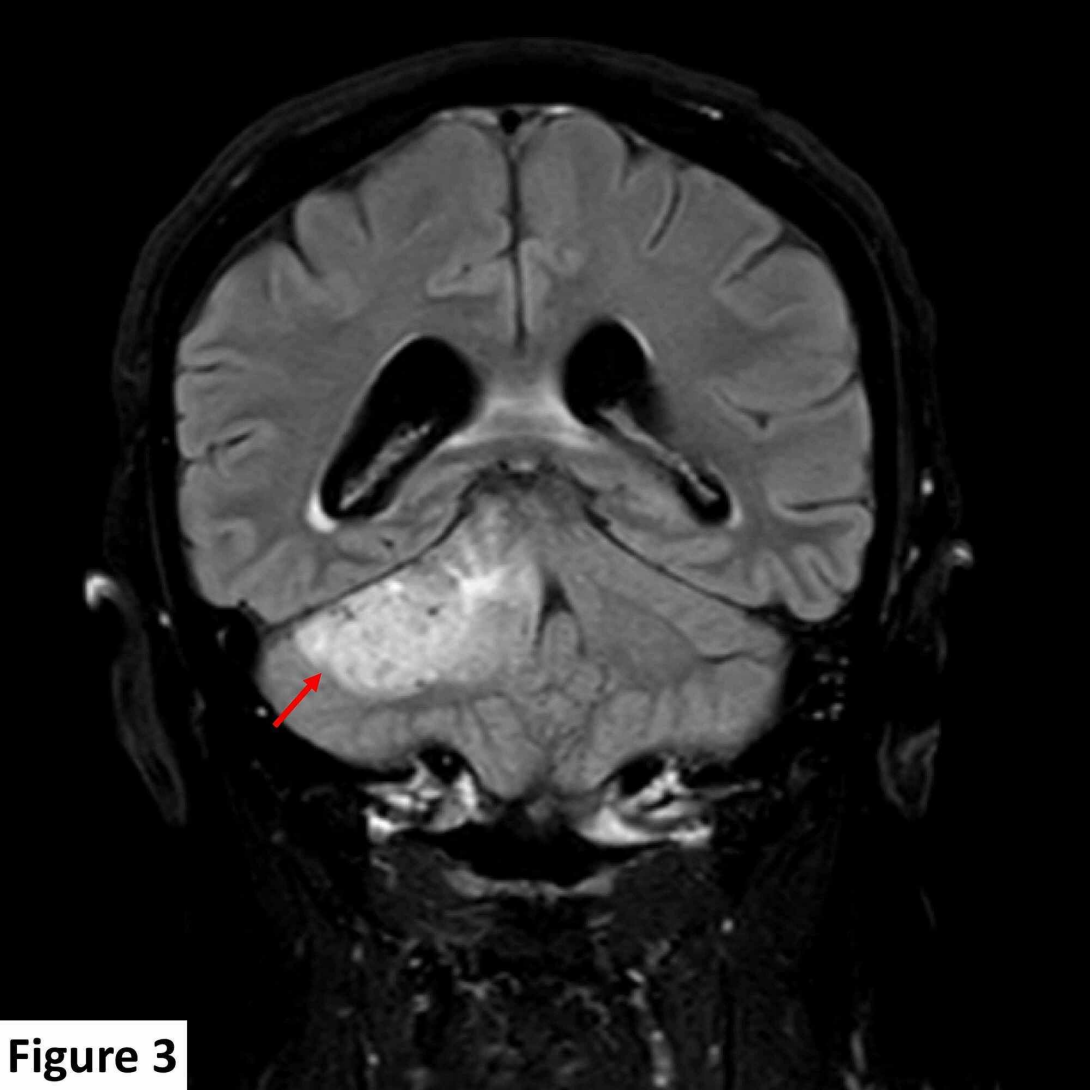
MRI scan at presentation. Coronal T2 FLAIR image shows a right cerebellopontine angle hyperintense extra-axial dural-based lesion (red arrow). MRI, magnetic resonance imaging; FLAIR, fluid attenuated inversion recovery

A brain magnetic resonance imaging (MRI) scan detected a 25 mm × 24 mm dural-based, extra-axial, T1 hypointense, T2 heterogeneously hyperintense right CPA lesion. The lesion was predominantly solid with minor cystic components and few vascular flow voids and demonstrated diffuse contrast enhancement post gadolinium administration. The lesion was indenting the right side of the pons and right middle cerebellar peduncle, inciting parenchymal T2 signal change consistent with edema, and displayed a broad base against the right tentorium cerebelli. The internal auditory canal was spared with no identifiable soft tissue, enhancement, or expansion. The seventh and eighth cranial nerve complexes were unremarkable (Figures 1-3).

**Figure 4:**
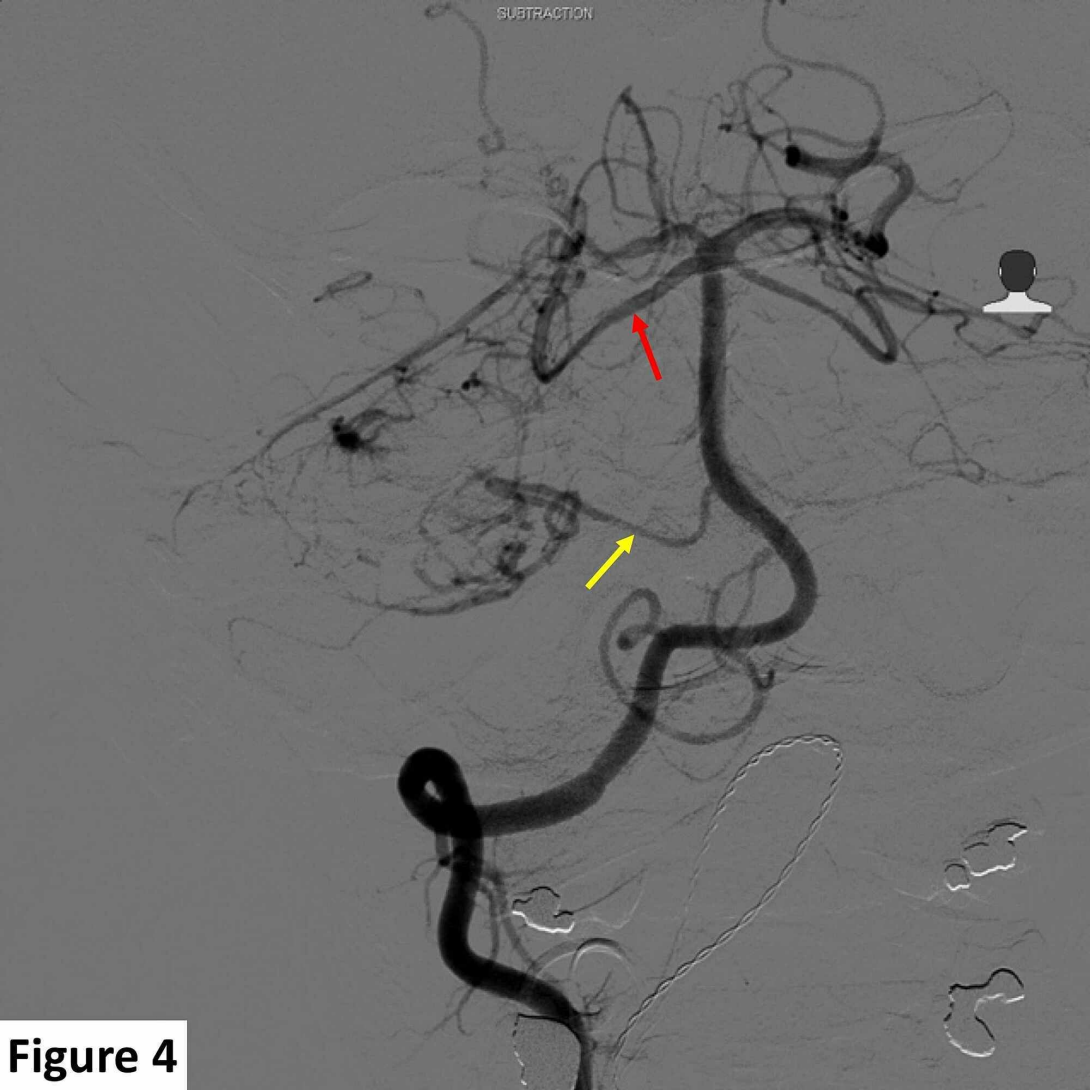
Preoperative right vertebral angiogram demonstrating lesional arterial supply. The majority of arterial supply is delivered from the right superior cerebellar artery (red arrow) while the anterior inferior cerebellar artery (yellow arrow) also contributed to the arterial supply of the lesion.

**Figure 5:**
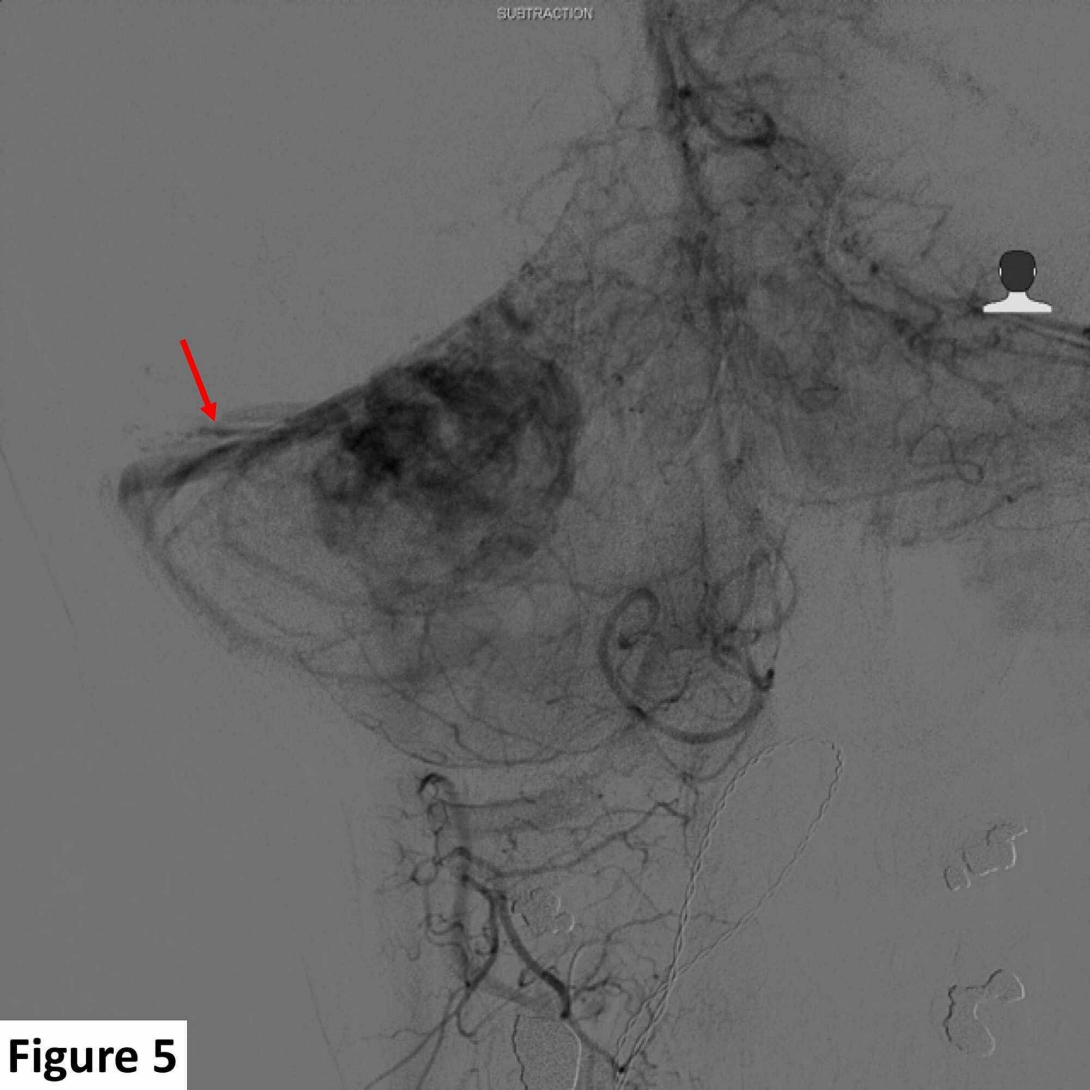
Preoperative right vertebral angiogram demonstrating lesional venous drainage. Venous drainage is along the right tentorium and into the right sigmoid sinus (red arrow).

The appearance was atypical for a dural-based meningioma or a schwannoma. A contrast-enhanced computed tomography scan of the chest, abdomen, and pelvis was unremarkable, excluding dural metastasis as a possibility. Due to the apparent vascular nature of the lesion and mass effect, a digital subtraction angiogram to map the vascular supply and plan preoperative tumor embolization was carried out. Detailed cerebral angiography demonstrated predominant tumoral arterial blood supply from the right superior cerebellar and right anterior inferior cerebellar arteries. Venous drainage was observed along the right tentorium and into the right sigmoid sinus (Figures 4, 5).

Superselective angiography and partial embolization of the tumor with N-butyl cyanoacrylate 15% was performed. Complete and adequate surgical resection followed and samples were sent for histological evaluation.

**Figure 6:**
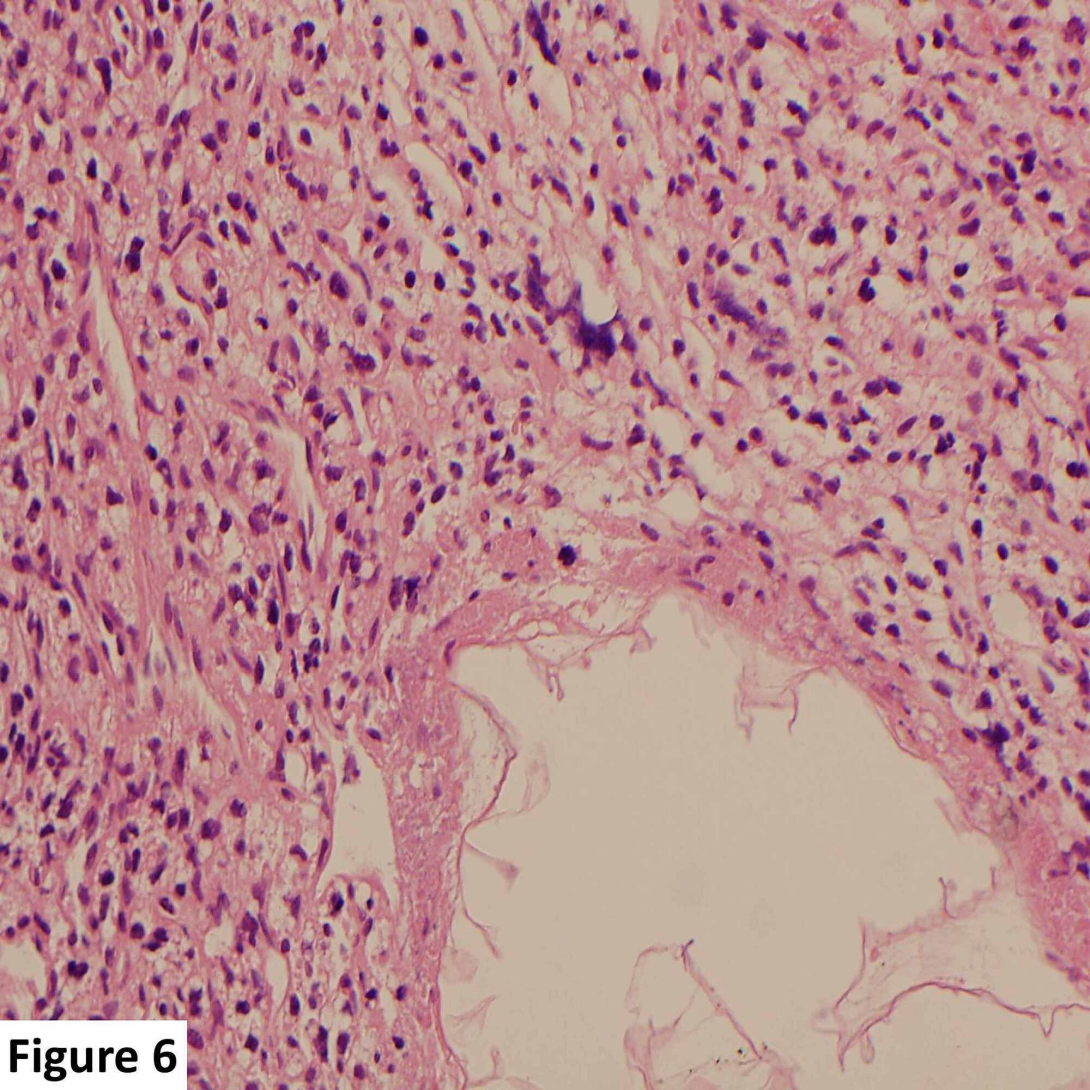
High-power magnification histological slide. Tumor cells have an abundant clear vacuolated cytoplasm, small uniform nuclei, and an interspersing rich vascular network. No areas of mitoses, hemorrhage, or necrosis are present.

**Figure 7:**
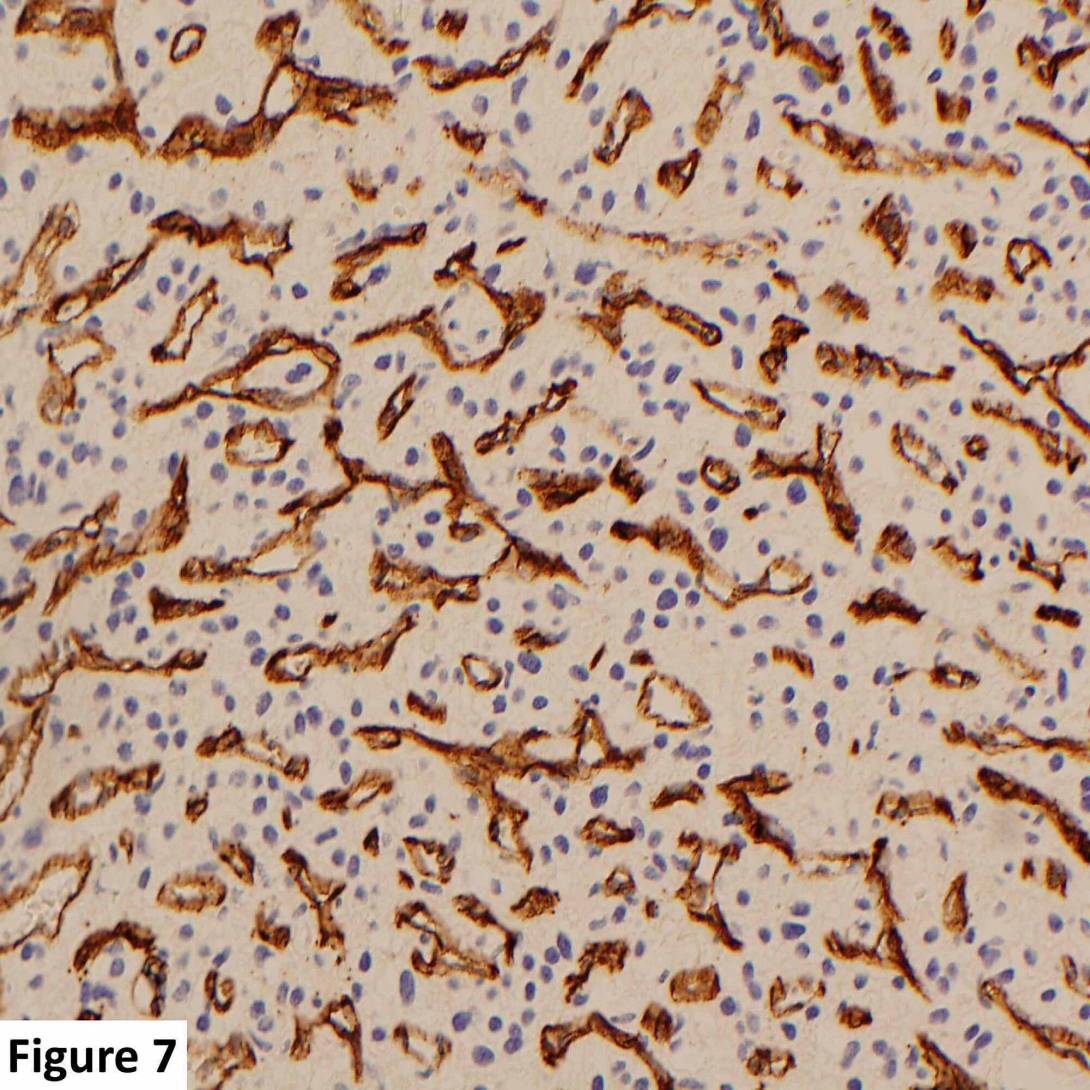
High-power magnification histological slide. Immunohistochemical CD34 staining is decorating the endothelial cells of the rich vascular network (brown color). The pale negative cells are the actual neoplastic stromal cells.

Histology and immunohistochemistry revealed a richly vascular tumor with polygonal clear stromal cells between thin-walled vascular channels which are decorated by the CD34 immunostain. The stromal cells were negative for cytokeratin (MNF116) and epithelial membrane antigen, thereby excluding a metastatic carcinoma. The tumor showed large areas of degeneration and necrosis while some of the larger feeding blood vessels showed intraluminal embolic material consistent with preoperative embolization. The histological diagnosis was that of an HB (Figures 6, 7).

**Figure 8:**
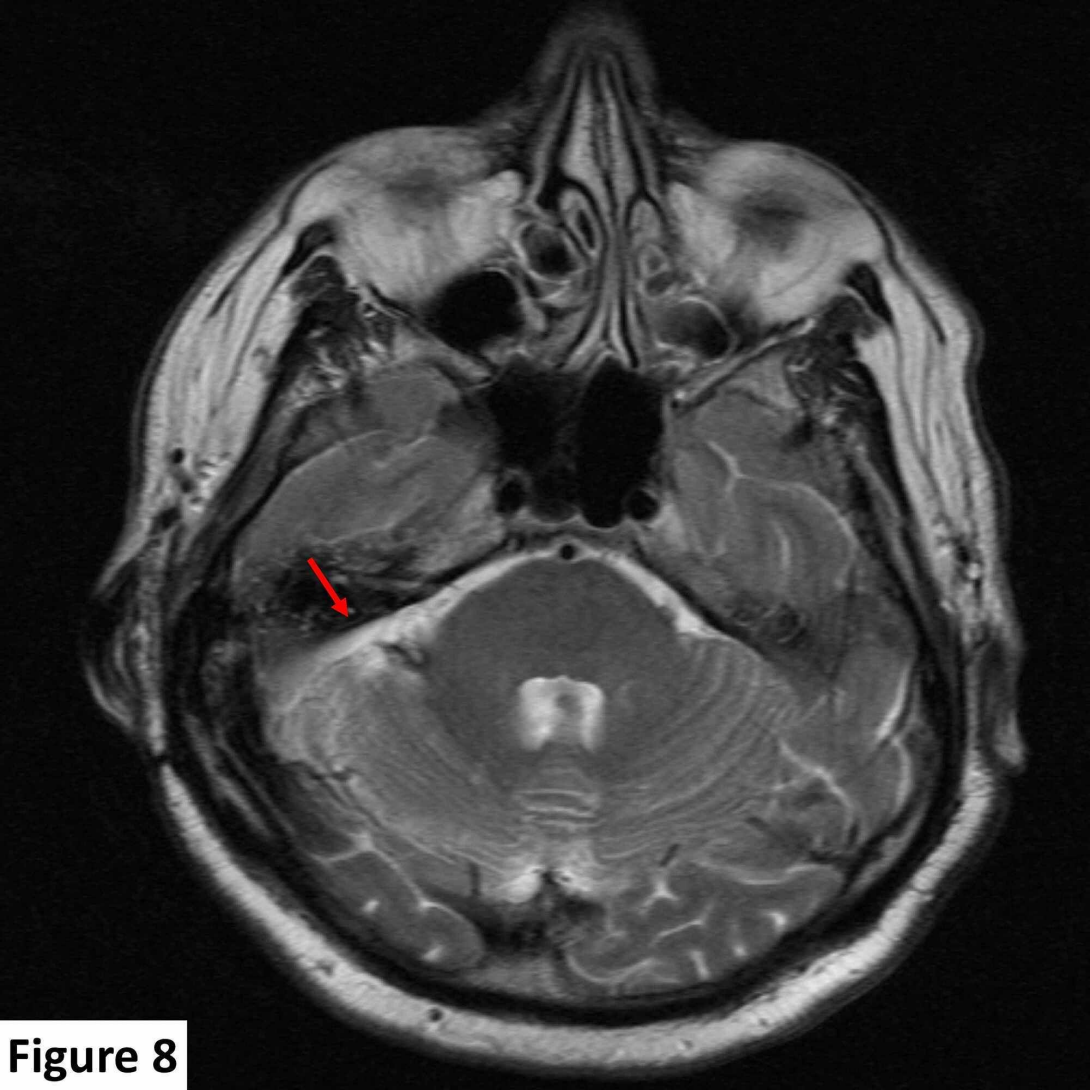
Follow-up axial T2 sequence MRI image. At 12 months postoperative follow-up there was no evidence of tumor recurrence (red arrow). MRI, magnetic resonance imaging

Because the tumor was solitary and there was no supportive family history, genetic testing for von Hippel-Lindau (VHL) syndrome was not carried out. A follow-up MRI scan 12 months postoperatively showed no tumor recurrence (Figure 8).

## Discussion

HBs are classically intra-axial benign WHO grade I hypervascular tumors of mesodermal origin. These lesions represent 2.5% of all intracranial tumors and up to 12% of all posterior fossa neoplasms [1]. The overall incidence of intracranial HBs is 0.15 per 100,000 person-years showing a peak incidence in the third to fifth decade of life and express a slight male predominance [2]. HBs are generally sporadic but can be associated with genetic-related conditions. Approximately 25% of cases are associated with VHL disease while 60% to 84% of VHL disease patients have HBs [3].

Tumors of the CPA constitute 6% to 10% of all intracranial neoplasms. Most are vestibular schwannomas and meningiomas but as many as 20% are of other etiologies [4]. Extra-axial CPA HBs are very rarely described in the literature and their presence poses a diagnostic and management challenge [5-7]. To explain such rare extra-axial imaging characteristics, it has been suggested that HBs, being derived from cerebellar pial vessels, adopt an initial intra-axial nidus. However, with subsequent growth, lesional progression is directed into the extra-axial CPA and not into the cerebellar parenchyma [8]. With extra-axial imaging characteristics, these lesions mimic the more common CPA pathologies (especially vestibular schwannomas) making correct identification a challenge due to similarities in MRI appearances [6,9,10]. Furthermore, correct diagnosis of such lesions is of pivotal importance from a surgical planning point of view. HBs excision in the CPA is a demanding task due to tumor vascularity, narrow surgical corridor, and the requirement for circumferential dissection with the proximity of the brainstem and cranial nerves [11].

MRI is the modality of choice in scrutinizing extra-axial CPA lesions which can be of vascular, meningeal, or cranial nerve origin [12]. Vascular lesions include vertebrobasilar aneurysms which appear as enhancing, rounded, and well-defined T2 hypointense lesions. The most prevalent among all extra-axial CPA lesions are meningiomas and vestibular schwannomas [4]. Meningiomas are classically smooth, possibly calcific, extra-axial lesions that express a characteristic dural tail. Vestibular schwannomas and HBs can be challenging to differentiate due to their MRI similarities. Both are T1 iso-hypointense, T2 hyperintense, and enhance strongly with gadolinium. Classically, HBs appear as large, cystic, well-circumscribed masses with a hypervascular enhancing mural nodule and little or no surrounding parenchymal edema. Less often, HBs present as solid lesions. HBs, unlike vestibular schwannomas, do not usually involve the internal auditory canal. The vascular nature of HBs manifests as a prolonged tumor blush on MR angiography and as flow voids on conventional MRI. On the other hand, vestibular schwannomas typically involve the internal auditory canal and cause widening of the porusacousticus, a feature not typically seen in HBs. However, intracisternal schwannomas may be heterogeneously hypervascular mimicking HBs on MRI [12,13].

HB is best managed in a multidisciplinary fashion by interventional neuroradiologists, neurosurgeons, radiation oncologists, and neurooncologists. Management options include surgical resection, radiotherapy, and to some extent the use of endothelial growth factor inhibitors. Surgical resection offers definitive therapy for sporadic HBs, particularly those arising in the cerebellum. The role of surgery in patients with VHL disease is less well defined due to the frequent occurrence of additional synchronous and metachronous lesions. Due to their high vascularity, HBs frequently require angiographic evaluation to identify feeding arteries with subsequent preoperative embolization of such feeders using polymer microspheres, ethanol, or polyvinyl alcohol particles. Furthermore, preoperative radiosurgery has shown to reduce the vascularity and control bleeding of this hypervascular neoplasm prior to resection [14]. Such an approach allows complete surgical resection with acceptable morbidity [15]. Although the optimal timing of surgery is uncertain, the consensus is initial MRI evaluation. If there is any symptomatology, neurological deficit progression, evidence of tumor or cyst growth, or hemorrhage, operative intervention should be considered. The role of stereotactic radiotherapy, particularly in VHL patients with multiple tumors and those with surgically inaccessible lesions, has been significantly evaluated with various publications reporting a five-year tumor control rate of 82% to 94% [16,17]. Because of the role of vascular endothelial growth factor in the genesis of HBs, the role of such antiangiogenic agents has been evaluated. In patients with VHL disease, pazopanib demonstrated a partial response in 4% of those with HBs and stabilization of disease in the majority of patients, but resulted in intracranial hemorrhage in 7% of the cases. On the other hand, sunitinib failed to demonstrate an acceptable clinical response [18,19]. Although patients with sporadic disease usually exhibit a disease-free postoperative course, patients with VHL disease are more likely to have local recurrence. Here, regular long-term follow-up is recommended to detect local and distant recurrence, even if the clinical course is benign and the tumor is totally resected [20].

## Conclusions

Extra-axial HBs are rarely encountered highly vascular intracranial lesions that should be included in the differential diagnosis of CPA tumors. While uncommon, the potential for misdiagnosis and inappropriate surgery could be disastrous due to the potential for catastrophic bleeding from these tumors. MRI plays an important role in depicting the lesional imaging characteristic and distinguishing HBs from other CPA tumors, particularly vestibular schwannomas. Management is delivered in a multidisciplinary fashion with treatment options being tailored to fit the patient’s clinical circumstances. Published evidence suggests that although HBs of the CPA are challenging lesions to treat surgically, these lesions can be removed safely when they are appropriately diagnosed. Other management options include radiotherapy (particularly in VHL patients), and to some extent, the use of endothelial growth factor inhibitors. Patients with VHL disease are more likely to have a local recurrence. For this reason, regular long-term follow-up is recommended to detect local and distant recurrence.
